# New Insights into Immune-Based Diagnosis, Therapy and Prophylaxis for Infectious Diseases 2020

**DOI:** 10.1155/2021/9767464

**Published:** 2021-12-30

**Authors:** Giuseppe A. Sautto, Roberta A. Diotti, Kristen M. Kahle

**Affiliations:** ^1^Center for Vaccines and Immunology, University of Georgia, Athens, GA 30602, USA; ^2^Laboratory of Medical Microbiology and Virology at “Vita-Salute” San Raffaele University, 20132 Milan, Italy; ^3^Pomona Ricerca S.r.l., Turin, Italy; ^4^Spark Therapeutics, Inc., Philadelphia, PA, USA

Immune-based diagnostic, therapeutic, and prophylactic tools have played a central role in medicine since the discovery of antibodies at the end of the 19^th^ century.

Since then, more and more sophisticated antibody-based approaches have been developed allowing to easily diagnose different types of disorders spanning from infectious diseases to premalignant, malignant, and autoimmune diseases.

As an example, the enzyme-linked immunosorbent assay (ELISA) represents one of the simplest but still most powerful methods for the diagnosis of different types of diseases, and thanks to its versatility, multiple formats have been developed such as the competitive ELISA, the sandwich ELISA, and the indirect ELISA. Additionally, besides assessing the binding to an antigen, antibody-based method approaches can also have a prognostic value since they can assess the presence, for example, of neutralizing antibodies eliciting a protective effect. In this regard, the hemagglutination inhibition assay (HAI) represents the gold standard method to evaluate the efficacy of not only current standard of care but also underdevelopment next-generation influenza vaccines in eliciting a neutralizing and protective immune response. As for ELISA, the HAI has also been developed in different formats in order to dissect the antibody response, for example, following influenza infection or vaccination. In this context, our group recently described a competitive HAI-based assay using a combination of influenza virus and recombinant influenza hemagglutinin (HA) proteins to dissect the HAI functional activity of HA-specific antibody populations in a single assay format [1].

In parallel to their ease of manufacturing and deployment as highly specific molecules, antibodies represent also a fundamental tool for the development of prophylactic and therapeutic immune-based strategies. As an example, in this special issue, F. Norouzi et al. [2] described the use of egg yolk-specific antibodies (IgY) raised against the outer membrane protein F (OprF) of *Pseudomonas aeruginosa* in a murine burn model of infection. Importantly, immunotherapy with anti-OprF IgY resulted in a significant improvement in the survival of mice infected by *P. aeruginosa* and this phenomenon has been mechanistically confirmed *in vitro* using a A549 cell-based invasion assay. This work represents a further demonstration of the efficacy of antibody-based approaches in limiting infectious diseases. Additionally, thanks to the discovery of methods for developing monoclonal antibodies (mAbs), immune-based therapies are today routinely used in the context of autoimmune and oncologic diseases and are starting to be used more frequently in the clinical practice for the treatment of other types of disorders such as infectious diseases. The current COVID-19 pandemic has further demonstrated, thanks to the extraordinary advancements in the field, the faster discovery and effectiveness of antibody-based approaches as a rapid first-line developed antiviral molecules against SARS-CoV-2, as mentioned by H. Ouassou et al. in their review article of this special issue [3]. In fact, soon after the pandemic outbreak, a plethora of mAbs were soon described in the literature with some of them concomitantly entering in the clinical trial *iter* [4, 5].

In addition to their direct role as immunotherapeutic and immunoprophylactic molecules, antibodies play also a pivotal role as indirect tools to dissect the antibody response following infection or vaccination. In this context, our group recently described the development of mAbs against Computationally Optimized Broadly Reactive Antigens (COBRA) for influenza as a way to dissect the determinants of the humoral response and shed light on the mechanisms responsible of the broad antibody-mediated neutralization and protection conferred by COBRA-based influenza vaccines [6].

In addition to the B cell arm-based tools, the T cell-mediated components of the immune response play a central role not only in the context of our immunity but also as a valid instrument to develop diagnostic and prophylactic tools against infectious diseases. As an example, in this special issue, L. M. Elamin Elhasan et al. described through the use of immunoinformatic approaches the prediction of the most conserved and immunogenic B and T cell epitope peptides of the fructose bisphosphate aldolase (Fba1) for the development of a *Candida glabrata* vaccine [7]. Similarly, M. Okutani et al. screened the CD4+ and CD8+ T cell epitopes in the gH/gL/gQ1/gQ2 tetrameric complex protein of the human herpesvirus 6 subtype B (HHV-6B) in a mice immunization model [8]. In particular, this group identified multiple CD4+ and CD8+ T cell-stimulating peptides both in BALB/c and C57BL/6 mouse strains, highlighting the potential of the gH/gL/gQ1/gQ2 tetramer-targeted strategy for the future development of T cell-based vaccine and immunotherapies against HHV-6B.

In this special issue, all these aspects are covered by two review articles and four research papers discussing how we can exploit and utilize the immune system to understand new host-pathogen relationships as well as for the development of novel prophylactic, therapeutic, and diagnostic tools. We hope that the readers of this special issue will appreciate the interesting findings and the reviewed concepts of the field discussed in these papers.

*Giuseppe A. Sautto* *Roberta A. Diotti* *Kristen M. Kahle*
